# Sarcopenia and lower urinary tract diseases: links, mechanisms, and clinical implications

**DOI:** 10.3389/fnut.2025.1704456

**Published:** 2025-11-25

**Authors:** Shanyu Liu, Hongliang Cao, Liming Wang, Shuxin Li, Yuwei Liang, Yubo Feng, Zihan Gao, Song Wang, Xin Lian

**Affiliations:** Department of Urology II, The First Hospital of Jilin University, Changchun, China

**Keywords:** sarcopenia, lower urinary tract diseases (LUTDs), pelvic floor dysfunction, resistance exercise, therapy, frailty, neoplasms, pelvic floor

## Abstract

Lower urinary tract diseases (LUTDs), including lower urinary tract symptoms (LUTS), overactive bladder (OAB), urinary incontinence (UI), bladder cancer (BC), prostate cancer (PCa), and benign prostatic hyperplasia (BPH), severely impair the quality of life of the elderly. Emerging evidence highlights a strong association between sarcopenia (progressive loss of muscle mass, strength, and function) and the prevalence, severity, and progression of LUTDs, as well as poorer treatment responses in affected patients—though most supporting studies are cross-sectional or retrospective, with prospective trials needed to confirm causality. Potential mechanisms linking sarcopenia to LUTDs include pelvic floor muscle weakening, neuromuscular dysfunction, metabolic/endocrine disturbances, genetic factors, and gut microbiome dysregulation. Clinically, interventions such as resistance exercise, nutritional support, gut microbiome-targeted strategies, pelvic floor training, and pharmacological therapies show promise in mitigating LUTDs symptoms by targeting sarcopenia. Integrating sarcopenia assessment into LUTDs management could improve patient care; future research should prioritize large-scale prospective trials to validate causal relationships, clarify key mediating mechanisms (e.g., specific gut microbial taxa, neuromuscular signaling pathways), and develop personalized intervention protocols tailored to distinct LUTD subtypes and patient characteristics.

## 1 Introduction

Lower urinary tract diseases (LUTDs)—including lower urinary tract symptoms (LUTS), overactive bladder (OAB), urinary incontinence (UI), bladder cancer (BC), prostate cancer (PCa), and benign prostatic hyperplasia (BPH)—severely reduce quality of life in older adults (1). Current management strategies (pharmacotherapy, behavioral interventions, and surgery) alleviate symptoms to some extent, but many patients still experience persistent symptoms, suboptimal treatment responses, or adverse effects. This highlights the need for complementary approaches.

Sarcopenia is a progressive, generalized skeletal muscle disorder defined by loss of muscle mass, strength, and function. Clinically, it presents as reduced muscle strength, impaired physical performance, and higher fall or frailty risk, particularly in older adults (2). The latest diagnostic criteria (from the European Working Group on Sarcopenia in Older People 2, EWGSOP2) prioritize low muscle strength, with low muscle quantity/quality and poor physical performance supporting severity grading (3). Recent studies have linked sarcopenia to a range of systemic diseases, and growing observational evidence (cross-sectional and retrospective studies) points to a strong association with LUTDs.

To inform this narrative review, we searched PubMed for studies combining “sarcopenia” with “OAB,” “LUTS,” “BC,” “PCa,” or “UI,” retrieving 291 articles as of October 24, 2025. We screened these articles in two stages: first excluding those unrelated to the keywords, then filtering by three themes (association analysis, mechanisms of action, and therapeutic strategies) to select studies relevant to our focus.

This review synthesizes current evidence on the sarcopenia-LUTDs relationship, explores potential underlying mechanisms, and discusses the clinical value of targeting sarcopenia in LUTDs management. Our goal is to provide new insights to guide improvements in clinical outcomes for patients with these prevalent conditions.

## 2 A substantial body of observational evidence supports a strong link between sarcopenia and lower urinary tract diseases

### 2.1 Sarcopenia and lower urinary tract symptoms

LUTS are a series of symptoms caused by dysfunction of the bladder, urethra, and its neuromodulatory system, and are usually categorized as storage, voiding, and postvoiding symptom. A significant association between sarcopenia and LUTS has been noted in the literature. In a cross-sectional study, researchers analyzed data using 377 women and 264 men over the age of 70 and concluded that skeletal muscle health, physical performance, and LUTS were negatively associated (4). In a study based on data from the 2005–2006 National Health and Nutrition Examination Survey (NHANES), which included 959 men aged 40 years and older (mean age 52.08 ± 7.91 years), low lean body mass, a key indicator of sarcopenia, was found to be significantly associated with an increased risk of developing lower LUTS after weighted multivariate regression analyses adjusting for the confounding factors of age and body mass index. There was a significant association. Specifically, low lean body mass was associated with a significantly increased risk of urinary hesitancy, dysuria, dyspareunia, urinary frequency, daytime LUTS, and clinical LUTS, suggesting that sarcopenia may be a potential risk factor for LUTS in males, and that early intervention for loss of lean body mass may help to ameliorate LUTS (5). In a study of community-dwelling older men, cross-sectional analyses (including 352 men aged 65–97 years) showed that individuals with higher thigh muscle strength and specificity had significantly lower LUTS severity, providing a side note that sarcopenia may be associated with LUTS (6). A study by Hashimoto et al. demonstrated a significant association between sarcopenia, as evidenced by decreased psoas muscle index (PMI), and visceral obesity, as evidenced by elevated visceral fat area, and severe urinary storage symptoms in elderly female patients aged ≥65 years (7). In addition, sarcopenia can also affect the treatment outcomes of LUTS. In a study of 59 male patients aged ≥75 years who were receiving LUTS medication and had not had their medication regimen adjusted at 1 year, sarcopenia, defined by a SARC-F score (Sarcopenia Assessment Form) of ≥4, was shown to be significantly more ineffective in the treatment of LUTS medication in patients with this type of sarcopenia, with improvement in voiding symptoms being particularly deficient (8).

### 2.2 Sarcopenia and overactive bladder

OAB is a common lower urinary tract disease characterized by a sudden, involuntary contraction of the bladder muscle (detrusor muscle), leading to urgency, with or without urge incontinence, usually accompanied by frequency and nocturia, in the absence of urinary tract infection or other obvious pathology (1). Numerous studies have confirmed a significant association between sarcopenia and OAB: the prevalence of OAB is higher in patients with sarcopenia, and the severity of OAB symptoms is positively correlated with an increased risk of sarcopenia. A cross-sectional study involving 329 elderly diabetic patients aged ≥65 years revealed a significant association between sarcopenia and OAB in elderly diabetic male patients (9). A population-based cohort study applied univariable logistic regression analysis to assess the association between sarcopenia and OAB symptoms. The study demonstrated a significant association between OAB and sarcopenia, with the prevalence of sarcopenia increasing as OAB severity escalates (10). In a retrospective study conducted in the United States, sarcopenia was found to be an independent risk factor for OAB in the US adult population, and a positive correlation was observed between sarcopenia and OAB prevalence. In addition, the study demonstrated that models constructed on the basis of sarcopenia can be used to predict the risk of OAB (11). Sarcopenia is not only strongly associated with the prevalence of OAB, but is also a contributing factor to the development of chronically untreated or refractory OAB into detrusor hyperreflexia with impaired contractility and ultimately to the development of underactive bladder (12, 13). A retrospective study examining the relationship between sarcopenia, frailty and pelvic organ prolapse (POP) in elderly women found that exacerbation of OAB symptoms may occur concurrently with sarcopenia of the pelvic floor muscle group as POP progresses. The study also found that estrogen deficiency may lead to skeletal muscle atrophy (sarcopenia) and pelvic organ abnormalities, such as bladder abnormalities. While the study did not directly state that sarcopenia can worsen OAB, these findings imply a potential pathophysiological association between the two conditions (14).

### 2.3 Sarcopenia and urinary incontinence

UI is defined as the involuntary leakage of urine, representing a common symptom of lower urinary tract dysfunction that significantly impacts quality of life. The main types of UI include stress incontinence, urge incontinence, mixed incontinence, overflow incontinence and functional incontinence. In a cross-sectional retrospective study that included 802 female subjects aged 60 years and older in a geriatric outpatient clinic, findings showed an independent association between low muscle mass and UI when muscle mass was adjusted for body weight or Body Mass Index (BMI), suggesting that sarcopenia may be associated with UI (15). In a study based on the 2011–2018 NHANES database, researchers explored the association between sarcopenia and UI in adult females under the age of 60. The study used an extremity skeletal muscle mass index (ASMI) < 0.512 as a diagnostic criterion for sarcopenia and showed that ASMI was significantly negatively correlated with UI and that the prevalence of UI was significantly higher in sarcopenic patients than in non-sarcopenic patients (16).

Not only for incontinence as a whole, sarcopenia has also been shown to correlate significantly with specific types of incontinence. In a cross-sectional analysis of Indian older adults, researchers explored the relationship between stress urinary incontinence (SUI) and sarcopenia. The results showed that sarcopenia was significantly positively associated with SUI in Indian older adults, and that this association was more pronounced in non-alcoholic men and women who had not undergone hysterectomy. Alcohol consumption and hysterectomy were both risk factors for urinary incontinence, whereas women who did not undergo hysterectomy and men who did not consume alcohol were among the excluded influences, which makes the direct link between sarcopenia and urinary incontinence more intuitive (17). Based on data from 11,168 adult women aged 20 years and older from the 2001–2006 and 2011–2018 NHANES in the United States, a study by Wang et al. found differences in the impact of decreased muscle mass in different body regions due to sarcopenia on SUI: ASMI was not associated with SUI risk, whereas a high trunk muscle index was associated with a significantly lower risk of SUI. This difference may be due to the nature of the ASMI, which reflects the ratio of skeletal muscle mass to BMI in the extremities, whereas the extremity muscles mainly maintain locomotion and limb strength, and their structure and function neither directly support the pelvic floor, nor are they involved in urinary control, nor do they directly regulate abdominal pressure (18). Lower trunk muscle mass index is an independent predictor of severe SUI (19). This finding not only helps to reduce the workload of skeletal muscle mass measurement in the clinic, but also provides guidance for targeted preventive exercise. And one article noted a significant negative correlation between ASMI and the risk of urge urinary incontinence (UUI), i.e., the higher the ASMI, the lower the risk of UUI (20). A cross-sectional analysis of 3,557 U. S. adult women aged 20 years and older based on data from the 2001–2004 NHANES showed that sarcopenia was significantly associated with an increased risk of mixed urinary incontinence (MUI); Further age-stratified analysis showed that sarcopenia was an independent risk factor for SUI in women aged 60 years and older, whereas sarcopenia was an independent risk factor for MUI in women aged 40–59 years (21). In a multicenter cross-sectional study of residents of five nursing homes, researchers found a significant association between the risk of sarcopenia and UI, with the highest prevalence of functional urinary incontinence (FUI) in this group (22). It has been suggested that by measuring calf circumference in the elderly population, it is possible to screen for sarcopenia and intervene in a timely manner to reduce the risk of incontinence (23).

Sarcopenia is associated not only with the development of UI but also with the recovery of voiding function. In a single-center retrospective cohort study of 917 patients in late-acute rehabilitation, results showed a significant negative impact of sarcopenia on patients’ recovery of independence in urinary and bowel function (24). Conversely, the more significant the improvement in sarcopenia (e.g., increased muscle mass, improved muscle strength), the better the recovery of voluntary control of urination and defecation (25). However, it has been shown that sarcopenia is not significantly associated with postoperative urinary incontinence in patients undergoing non-nerve-preserving robot-assisted radical prostatectomy (RARP). This may be due to the fact that the psoas major index was used as a criterion for assessing sarcopenia in this study, whereas simple loss of muscle mass does not seriously affect pelvic floor muscle function; whereas the other topic of this paper, muscle steatosis, is an important independent predictor of postoperative urinary incontinence because muscle steatosis is not a simple loss of muscle bulk, but rather due to a reduction in muscle tissue. Because muscle steatosis is not a simple loss of muscle volume, but rather a reduction in muscle strength due to an increase in the fat content of muscle tissue (i.e., a decrease in muscle contractility), it has a greater impact on pelvic floor muscle function (26).

### 2.4 Sarcopenia and bladder cancer

BC is a malignant tumor originating from the epithelial cells of the bladder mucosa and is one of the most common cancers of the urinary system, which is typically characterized by painless hematuria of the naked eye. Accumulating evidence demonstrates that sarcopenia adversely impacts the prognosis of muscle-invasive BC (MIBC), correlating with higher cancer-specific mortality (CSM) and overall mortality (OM) (27). This negative association extends across various treatment modalities, with sarcopenia emerging as a consistent predictor of poor outcomes. Understanding the impact of sarcopenia on perioperative muscle mass dynamics and outcomes may aid in preoperative rehabilitation and dose optimization.

Among patients undergoing radical cystectomy (RC), sarcopenia independently predicts reduced survival, including shorter overall survival (OS) and cancer-specific survival (CSS) (28). Yamashita et al. further demonstrated that both sarcopenia and muscle steatosis were independent adverse predictors of CSS, with sarcopenia also associated with shorter OS (29).

Mayr et al. found sarcopenia to be an independent predictor of 90-day postoperative mortality (30). Psutka et al. (31) confirmed sarcopenia as an independent risk factor for CSM and all-cause mortality (ACM) in their multifactorial analysis, a finding subsequently replicated by Erdik et al. (32) and Mayr et al. (33) in larger cohorts. Sarcopenic patients also tend to have longer hospital stays, lower BMI, and poorer long-term survival rates (34). Similar trends are observed in non-muscle invasive BC (NMIBC) treated with transurethral resection and BCG therapy, where sarcopenia is linked to worse recurrence-free survival (RFS) and OS (35). Miyazaki et al. identified sarcopenia as an independent predictive factor for overall survival rates in MIBC patients undergoing RC (36). Ha et al. observed postoperative sarcopenia incidence increased from 32.5 to 50.0%, correlating with higher tumor stage (37). Pang et al. demonstrated sarcopenia was an independent risk factor for OS and progression-free survival (PFS) (38). Beyond survival outcomes, sarcopenia increases the risk of postoperative complications such as parastomal hernia (39).

The impact of sarcopenia extends to systemic therapies. In MIBC patients receiving neoadjuvant chemotherapy (NAC), sarcopenia after treatment independently associated with increased CSM and elevated nephrotoxicity risk (40, 41). Kasahara et al. reported shorter OS in sarcopenic metastatic urothelial cancer patients receiving gemcitabine-nedaplatin (42). Platinum-based NAC exacerbated sarcopenia, with SMI declines and incidence increases from 69 to 81% (43–45). Preoperative sarcopenia also predicted poorer response to intravesical BCG in NMIBC (46).

In BC patients, the development of sarcopenia is closely related to disease progression and treatment. A systematic review of five retrospective cohort studies (438 patients) by Hansen et al. showed that the prevalence of sarcopenia in these patients ranged from 25 to 69% before treatment; it then increased significantly between 3 and 12 months of treatment, reaching 50 to 81% post-treatment. This suggests that BC treatment may exacerbate the development of sarcopenia (47).

Possible reasons why sarcopenia may lead to a poorer prognosis and treatment outcome for bladder cancer include: 1. Patients with sarcopenia have lower physical reserves or are unable to choose the best treatment option because of limited physical reserves (29); 2. Sarcopenia may affect the patient’s immune function and weaken the effect of anti-tumor therapy (35); 3. Sarcopenia may increase the likelihood of life-threatening diseases (e.g., fatal heart failure, respiratory disease, and cerebral infarction) after RC surgery (36). However, these conclusions are mostly inferences and lack of experimental data support. From the perspective of long-term research needs, in-depth excavation of the deeper mechanisms is still indispensable, and only in this way can we effectively fill the current gaps in the interpretation of specific mechanisms.

### 2.5 Sarcopenia and prostatic disease: prostate cancer and benign prostatic hyperplasia

PCa is a malignant tumor that develops in the prostate gland, a small walnut-shaped organ in males responsible for producing prostatic fluid (a key component of seminal fluid). It is one of the most common cancers in men, particularly in older age groups.

In recent years, a large number of systematic reviews and meta-analyses have explored the relationship between sarcopenia and prostate cancer (PCa). Among them, the study by de Pablos-Rodríguez et al. rigorously included 9 longitudinal observational studies involving a total of 1,659 patients, and clearly identified sarcopenia as an independent poor prognostic factor for progression-free survival (PFS) in patients with advanced PCa (multivariate hazard ratio [HR] = 1.61, 95% confidence interval [CI]: 1.26–2.06, *p* < 0.01) (48). This conclusion is supported by subsequent similar studie: Meyer et al. conducted a meta-analysis on the association between sarcopenia and survival in PCa patients (including 5 retrospective studies with a total of 1,221 patients), which further verified that in multivariate analysis, patients with low skeletal muscle mass (LSMM) defined by CT are an important adverse prognostic factor for overall survival (OS) in PCa patients. In univariate analysis, the pooled hazard ratio (HR) for the association between LSMM and OS was 1.4 (95% confidence interval [CI]: 0.7–2.5), and in multivariate analysis, the pooled HR was 1.6 (95% CI: 1.2–2.1) (49). Moreover, sarcopenia affects prostate cancer in multiple aspects, which will be introduced one by one in the following paragraphs.

First, sarcopenia affects the survival and prognosis of PCa patients. PCa patients with sarcopenia prior to cancer diagnosis have a significantly increased ACM, and this association is more pronounced in patients with longer expected survival (50). In patients with castration-resistant prostate cancer (CRPC) receiving cabazitaxel chemotherapy, those with sarcopenia had a significantly shorter median OS (5.45 months) compared to those without sarcopenia (16.82 months), making it an independent factor for poor prognosis (51). In patients with metastatic castration-resistant prostate cancer (mCRPC), sarcopenia is an independent risk factor for poor prognosis following docetaxel treatment, with significantly shorter survival (52). In patients with metastatic hormone-sensitive prostate cancer (mHSPC), most have sarcopenia, which is associated with shorter CSS and OS, particularly in patients under 73 years of age. It is also associated with shorter prostate-specific antigen (PSA) progression time and treatment failure time (53, 54). Patients with CRPC and severe sarcopenia have a lower PSA response rate to cabazitaxel treatment, but OS and PFS are not significantly different from those without severe sarcopenia (55).

Sarcopenia is also associated with the progression and functional outcomes of PCa. In patients with CRPC, low skeletal muscle mass is an independent adverse prognostic factor for disease progression, and sarcopenia is significantly associated with radiographic progression (aHR = 2.39) (56, 57). It also affects treatment efficacy. In patients with localized PCa receiving low-dose-rate brachytherapy with iodine-125, 37.4% had sarcopenia, and at 12 and 24 months post-treatment, their UCLA-PCI (University of California, Los Angeles Prostate Cancer Index) urinary function significantly deteriorated, with a higher likelihood of clinically significant urinary function decline (58).

Conversely, PCa treatment can also induce or exacerbate sarcopenia. Non-metastatic PCa patients undergoing androgen deprivation therapy (ADT) experience a significant decrease in lean body mass, with a more pronounced decline observed in patients aged 70 years or older and those treated for ≤6 months (59); After 36 weeks of maximum ADT, the average total lean body mass decreased by 2.4%, with more pronounced decreases in limb lean body mass (upper limbs 5.6%, lower limbs 3.7%) (60). Advanced PCa patients receiving different ADT regimens (LHRHa monotherapy, LHRHa + abiraterone, LHRHa + enzalutamide) all experienced significant reductions in skeletal muscle mass, with combination therapy resulting in more pronounced muscle loss at 6 months compared to monotherapy (61). After 18 months of ADT combined with enzalutamide treatment, mHSPC patients experienced a 6.7% decrease in lean body mass and a 9.2% decrease in arm lean body mass index (62). High-risk PCa patients who received radiotherapy combined with ADT experienced an average decrease of 5.5% in SMI within 180 days, and those with an SMI decrease of ≥5% had a 5.6-fold higher risk of non-cancer mortality compared to those with stable SMI (63). In addition to ADT, other treatments may also cause or exacerbate sarcopenia. CRPC patients who received abiraterone monotherapy experienced a decline in muscle mass, with the most significant decline observed in those with a baseline BMI > 30 (64). Patients with mCRPC treated with abiraterone or enzalutamide both experienced significant muscle loss, with enzalutamide causing muscle loss to occur earlier (65). Patients with metastatic PCa who received ADT combined with other drugs experienced significant muscle loss (66); Among patients treated with abiraterone acetate + prednisone (AAP), 72.1% had sarcopenia, and SMI significantly decreased during treatment (67).

In terms of treatment, sarcopenia also affects the toxicity and efficacy of PCa therapy. Fully recognizing the impact of sarcopenia on perioperative muscle mass dynamics and outcomes may aid in preoperative rehabilitation and dose optimization. Regarding treatment efficacy, CRPC patients with severe sarcopenia had significantly lower PSA response rates to cabazitaxel therapy, and the PMI was an independent predictor of PSA response (55). Regarding treatment toxicity, among mCRPC patients receiving docetaxel therapy, 59.1% of those with sarcopenia and low muscle mass experienced dose-limiting toxicity (DLT), significantly higher than patients without these characteristics (68). In mCRPC patients receiving androgen receptor targeted therapy (ARAT), sarcopenia was significantly associated with severe treatment toxicity (aOR = 6.26) and an increased risk of first emergency department visit (aHR = 4.41) (57). Notably, among CRPC patients receiving first-line ARATs, 78% had sarcopenia, and those with sarcopenia had significantly longer PFS than those without sarcopenia (26.21 vs. 16.96 months), making sarcopenia an independent favorable prognostic factor for PFS, suggesting that sarcopenia may enhance the efficacy of ARATs (69).

In contrast to the above, in PCa patients who underwent RP, sarcopenia was not significantly associated with postoperative functional outcomes (urinary control, erectile function) or oncological outcomes (tumor staging, biochemical recurrence) and was not a predictor of RP outcomes (70, 71).

Although numerous studies have observed the impact of sarcopenia on prostate cancer, the specific mechanisms remain unclear. Further research is needed to elucidate the underlying causes, thereby guiding treatment strategies for prostate cancer patients with sarcopenia.

BPH is a non-cancerous enlargement of the prostate gland, commonly occurring in aging men. It results from the proliferation of both glandular and stromal cells in the transitional zone of the prostate, leading to compression of the urethra and LUTS. Yang et al. found through bidirectional Mendelian randomization analysis that sarcopenia is causally related to certain urinary tract diseases. Among them, sarcopenia increases the risk of BPH (72). There is insufficient evidence on the value of screening for sarcopenia in patients with BPH combined with LUTS and whether it can be used as a risk stratifier for surgical or pharmacologic treatment.

It is important to note that most of the above studies on the association between sarcopenia and LUTDs were cross-sectional or retrospective in design, with insufficient correction for confounding factors, and the causality and timeliness of the association has not been verified by prospective trials, which is a direction for future studies to focus on.

The observational evidence described above, which includes cross-sectional analyses, retrospective cohorts, and population-based studies, consistently confirms that sarcopenia is not only a comorbidity but also a potential risk factor for lower urinary tract dysfunction that affects the onset, severity, and outcome of diseases, such as OAB, UI, and urologic cancers. However, these associations alone do not explain how sarcopenia contributes to LUTDs. To translate these epidemiologic findings into targeted interventions, it is critical to unravel the underlying biological pathways, and only by clarifying the mechanisms linking muscle loss to pelvic floor muscle weakness, neuromuscular dysfunction, metabolic disorders, limited physical activity, genetic factors, or the gut microbiome will we be able to develop strategies to break the cycle between sarcopenia and LUTDs. Therefore, the multifactorial mediators of this relationship are explored below to establish a mechanistic framework.

## 3 Multiple potential mechanisms mediate the influence of sarcopenia and lower urinary tract diseases

**Figure 1:**
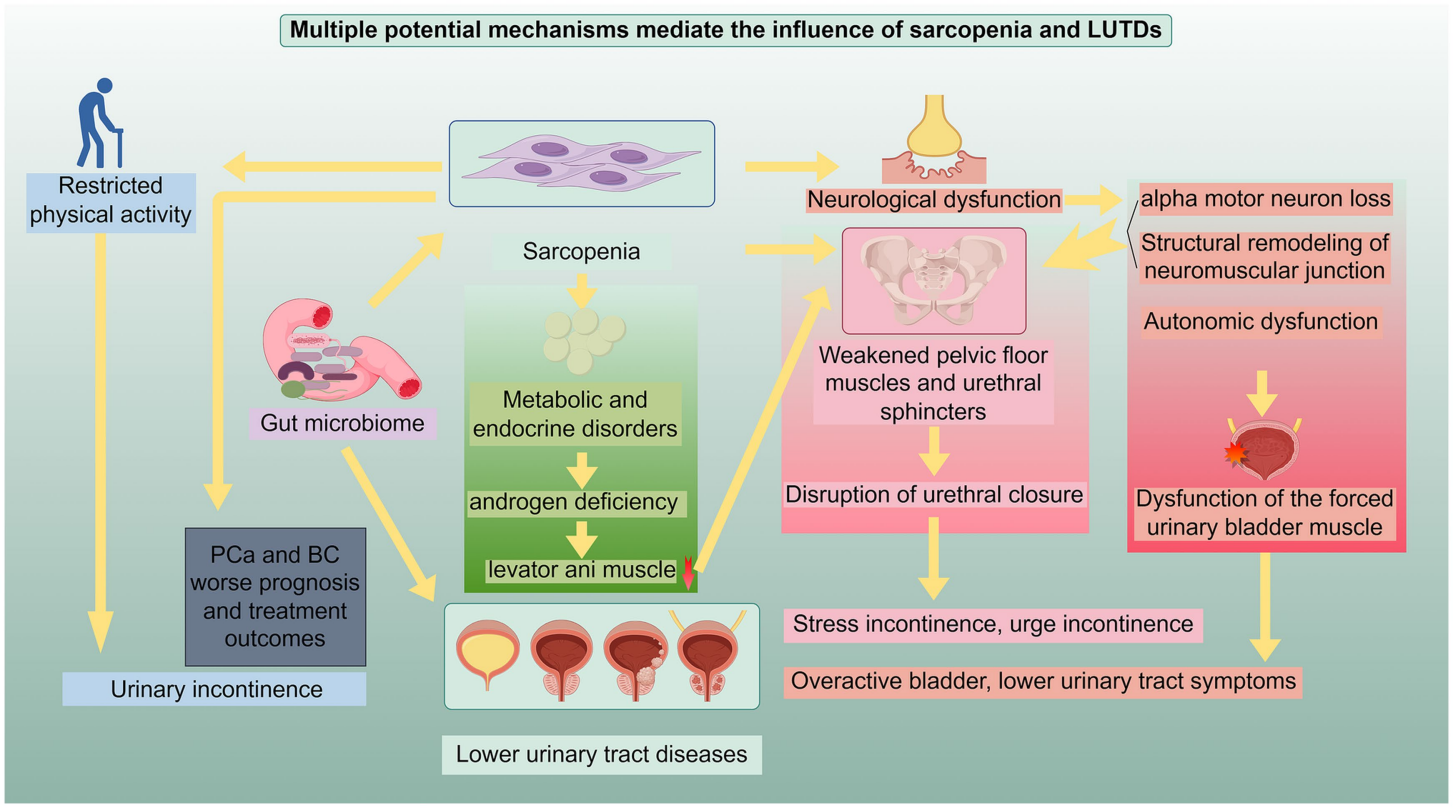
Potential mechanisms underlying the relationship between sarcopenia and LUTDs: This schematic illustrates the interrelated pathways through which sarcopenia and LUTDs exert reciprocal influences. The mechanisms include: (1) weakening of pelvic floor muscles and urethral sphincter function: Sarcopenia-induced atrophy and dysfunction of pelvic floor muscles and urethral sphincters disrupt urethral closure, leading to stress incontinence, urge incontinence, and other LUTD manifestations. (2) Neurological dysfunction: This encompasses alpha motor neuron loss, structural remodeling of neuromuscular junctions, and autonomic dysfunction, which impair the function of pelvic floor muscles, urethral sphincters, and detrusor muscles, contributing to overactive bladder, lower urinary tract symptoms, and detrusor dysfunction. (3) Metabolic and endocrine disorders: Androgen deficiency and other metabolic abnormalities in sarcopenia cause selective muscle loss (e.g., levator ani muscle), compromising pelvic floor support and linking to LUTDs. (4) Physical activity restriction and genetics: Sarcopenia-related restricted physical activity elevates the risk of urinary incontinence and worsens prognosis in prostate cancer (PCa) and bladder cancer (BC); shared genetic architecture (e.g., with benign prostatic hyperplasia) also underpins their association. (5) Gut microbiome dysregulation: Imbalanced gut microbiota in sarcopenia impacts PCa/BC prognosis and drives LUTDs via systemic inflammation and metabolic alterations. These mechanisms act independently or synergistically to mediate the bidirectional relationship between sarcopenia and LUTDs, highlighting pivotal targets for integrated clinical intervention. This figure was drawn by Figdraw.com.

Building on the observational evidence across LUTD subtypes—from overactive bladder (OAB), urinary incontinence (UI) and benign prostatic hyperplasia (BPH) to prostate cancer (PCa), lower urinary tract symptoms (LUTS) and bladder cancer (BC)—multiple interrelated pathways may link sarcopenia to lower urinary tract dysfunction (Figure 1). These mechanisms, which may act alone or in combination, include pelvic floor and urethral sphincter weakness (relevant to SUI and BPH-related LUTS), somatic and autonomic neuromuscular dysfunction (key to OAB and detrusor underactivity), endocrine-metabolic alterations [critical for PCa-related sarcopenia from androgen deprivation therapy (ADT)], reduced physical performance, shared genetic architecture (as seen in BPH), and gut microbiome dysregulation (implicated in BC and OAB; Table 1). While some of these pathways are supported by preliminary preclinical or clinical data, many remain partly hypothetical and require further experimental validation—particularly their subtype-specific roles (e.g., how androgen deficiency differs from genetic factors in driving sarcopenia-LUTD associations).

**Table 1:** Potential mechanisms and supporting evidence underlying the relationship between sarcopenia and LUTDs.

Potential mechanism (**Corresponding LUTDs**)	First author	Country	Years	Sample size	Subjects	Main findings	Reference
Weakening of the pelvic floor muscles and urethral sphincter function (UI/LUTS)	Douglass S. Hale	USA	1999	27	Human	Patients with genuine urinary incontinence exhibit significantly lower skeletal muscle content and proportion in the urethral sphincter, along with higher connective tissue content.	(86)
	Bag Soytas MD	Turkey	2021	92	Human	(1) Patients with stress urinary incontinence (SUI) exhibit significantly lower pelvic floor muscle strength compared to other incontinence subtypes. (2) Reduced grip strength may serve as a marker for pelvic floor muscle weakness. (3) The association between sarcopenia and urinary incontinence may be explained through the pathway: “low skeletal muscle strength → pelvic floor muscle weakness.”	(85)
	Monica Orsi Gameiro	Brazil	2012	51	Human	Urge urinary incontinence (UUI) Women with significantly weaker pelvic floor muscles Compared to stress urinary incontinence (SUI) Women	(88)
Neurological dysfunction (UI/OAB/LUTS)	Douglass S. Hale	USA	1999	27	Human	EMG findings in patients with genuine urinary incontinence reveal increased urethral sphincter fibrillation potentials, reduced motor unit action potentials, higher proportions of multiphasic waves, and lower maximal spontaneous electrical activity.	(86)
	Tsuyoshi Majima	Japan	2019	119	Human	Sarcopenia may impair detrusor contraction function by affecting detrusor smooth muscle function or autonomic innervation of the bladder.	(94)
	Verônica Porto de Freitas	Brazil	2018	76	Human	Sarcopenia is significantly associated with impaired parasympathetic modulation.	(95)
Metabolic and endocrine disorders (PCa/BC)	Jolanta Korczak	Poland	2023	64	Human	Long-term ADT combined with novel endocrine therapies may increase the risk of sarcopenia.	(66)
	Matthew R. Smith	USA, Canada, Finland,etc	2012	252	Human	Androgen deprivation therapy (ADT) is associated with persistent loss of skeletal muscle or lean body mass: LBM decreases by 1.0–2.4% over 1–3 years, with accelerated loss occurring as treatment duration increases.	(59)
	Ada S. Cheung	Australia	2019	63	Human	ADT therapy resulted in a significant decrease in total testosterone levels and selective muscle volume reduction—with the most pronounced decrease observed in the levator ani muscle.	(99)
Physical activity restriction and genetics (UI/BPH)	Wei-Ju Lee	China	2016	722	Human	Walking speed is an independent risk factor for UI (HR = 0.05 in multivariate logistic regression, 95% CI 0.003–0.987, *p* = 0.049).	(101)
	S. R. Bauer	USA	2023	3,235	Human	Increased individual frailty is nonlinearly positively correlated with the severity of LUTS.	(102)
	Feixiang Yang	China	2025	651,820	Human	Sarcopenia exhibits causal association with benign prostatic hyperplasia (BPH, OR = 1.17, *p* = 0.043). Both conditions demonstrate local genetic linkage in a specific region of chromosome 3 (3:139954597–141,339,097, *p* = 2.16E-05), with 75 shared risk genes identified. Multi-omics mediational analysis identified 17 metabolites and proteins (e.g., EFNB2, ALDOC) mediating their association, ultimately revealing shared genetic architecture between sarcopenia and BPH.	(72)
Gut microbiome (OAB/LUTS/BC/PCa)	Seung Yun Lee	Korea	2023	65	Mouse	The gut microbiota of sarcopenic mice exhibited altered abundance of *Alistipes*, *Lachnospiraceae*, and *Bacteroides*.	(104)
	Yilun Wang	China	2022	1,417	Human	In the gut microbiome of sarcopenia patients, seven bacterial species showed significantly increased abundance (e.g., *Desulfovibrio piger*, *Clostridium symbiosum*), with six of these positively correlated with sarcopenia severity.	(106)
	Roger A. Fielding	USA	2019	29 and 36	Human and Mouse	Patients with sarcopenia-related phenotypes exhibit significantly reduced abundance of the *Prevotellaceae* family, *Prevotella* genus, *Barnesiella* genus, and *Barnesiella intestinihominis* species in their gut microbiota, alongside a potential increase in pro-inflammatory bacterial communities such as the *Enterobacteriaceae* family.	(108)
	Teppei Okamoto	Japan	2021	1,113	Human	Among patients with urinary urgency, the relative abundance of *Bifidobacterium* was significantly reduced (2.41% vs. 4.23%, *p* = 0.014). The relative abundance of *Enterococcus faecalis* was significantly increased (9.25% vs. 6.26%, *p* = 0.006).	(110)
	Yoshiharu Okuyama	Japan	2022	669	Human	In patients with UU/UUI progression, the relative abundance of Streptococcus (a genus of bacteria harmful to human health) was significantly higher (3.8% vs. 2.3%, *p* < 0.001).	(111)
	Yijie Wang		2025	18,340	Human	Twelve specific gut microbial groups may have a causal relationship with PCa; the genera *Victivallis, Akkermansia, Odoribacter*, and *Butyrivibrio*, along with the families *Enterobacteriaceae* and *Verrucomicrobiaceae*, and the orders *Verrucomicrobiales*, *Enterobacteriales*, and *Verrucomicrobiae*, were positively correlated with PCa risk. Conversely, the *Eubacterium ruminantium* group, *Candidatus Soleaferrea*, and the UCG003 rumen bacterium family were negatively correlated with PCa risk.	(114)
	Sridhar Mani	USA	2024	-	Mouse	Gut microbiota metabolize N-butyl-N-(4-hydroxybutyl)nitrosamine (BBN) in the intestine into the bladder carcinogen N-n-butyl-N-(3-carboxypropyl)nitrosamine (BCPN).	(112)

UI, Urinary Incontinence; LUTS, Lower Urinary Tract Symptoms; OAB, Overactive Bladder; PCa, Prostate Cancer; BC, Bladder Cancer.

### 3.1 Weakening of the pelvic floor muscles and urethral sphincter function

The occurrence of LUTDs is closely related to weakened pelvic floor muscle strength and abnormal urethral sphincter function (73). These two factors interact through complex structural and functional associations to jointly affect the normal functioning of the urinary system (74). The pelvic floor muscles are an important structure that supports the bladder, urethra, and other pelvic organs. Studies have shown that pelvic floor dysfunction (PFD) is associated with sarcopenia (75, 76). Weakening of these muscles directly leads to insufficient support for the pelvic organs, causing abnormal bladder position or changes in the angle of the urethra, which in turn disrupts the urethra’s closure mechanism (77, 78). This weakening may be the result of muscle fiber atrophy and connective tissue replacement caused by aging, as well as muscle metabolic disorders triggered by a decrease in estrogen levels after menopause (79, 80). When the pelvic floor muscles are unable to provide sufficient support and contractile force, it becomes difficult to coordinate with the urethral sphincter to maintain urethral closure during urinary control (81).

The urethral sphincter, as a key structure in urine control, also plays an important role in LUTDs when its function is abnormal. The external urethral sphincter, composed of striated muscle, is responsible for active urinary control. A reduction in the number of muscle fibers, structural disorder, or decreased contractility can directly lead to insufficient urethral closure pressure (82). Dysfunction of the internal sphincter, composed of smooth muscle, may affect the basal tension and autonomic regulatory capacity of the urethra (83, 84). This functional abnormality may be related to muscle mass loss caused by sarcopenia, which prevents the urethra from closing effectively when abdominal pressure increases or from opening smoothly during urination, leading to problems such as UI or difficulty urinating (24, 75).

More importantly, the coordinated action of the pelvic floor muscles and the urethral sphincter is crucial during urination, and any imbalance between the two can further exacerbate LUTDs (74).

Specific to common LUTDs, studies have shown that stress incontinence is associated with weakened pelvic floor muscle strength (85, 86). Stress incontinence is most often due to a combination of inadequate pelvic floor muscle support and weakened contraction of the external urethral sphincter, which prevents the urethra from maintaining closure when abdominal pressure is elevated (87). Alternatively, urge incontinence may be associated with reduced pelvic floor muscle strength (88). However, the relevant studies are more sparse, and the possible mechanism is that urge incontinence may be associated with increased bladder sensitivity triggered by overactivity or spasm of the pelvic floor muscles. And the pelvic floor muscles may interfere with urinary function, leading to difficulty in urination or dysuria (89). A deeper understanding of these mechanisms may provide direction for clinical interventions to effectively prevent and treat LUTDs.

### 3.2 Neurological dysfunction

LUTDs may be associated with neurological factors. A study comparing urethral sphincter biopsy and electromyography (EMG) results between 17 women with normal urinary control and 10 women with genuine stress urinary incontinence (GSI) found that GSI women had significantly reduced skeletal muscle content and a higher proportion of connective tissue in their urethral sphincters, EMG showed more fibrillation potentials, fewer motor unit action potentials, and a higher proportion of multiphasic waves. These differences support the notion that neurogenic factors influence true stress incontinence (86).

Loss of alpha motor neurons as well as structural remodeling of the neuromuscular junction are important causes of sarcopenia, which in turn can continue to be exacerbated with loss of alpha motor neurons as well as structural remodeling of the neuromuscular junction (90). Dysfunction of the neuromuscular junction can hinder the conversion of nerve signals into muscle contractions effectively (91), which may weaken the pelvic floor muscles’ support function and the sphincter muscles’ closing ability. However, direct evidence from studies proving that sarcopenia weakens these muscles through neuromuscular junction dysfunction is still lacking, and further research is needed. At the same time, patients with sarcopenia have fewer myosatellite cells, which weakens muscle repair and further aggravates muscle atrophy (92, 93). Dysfunction of the pelvic floor muscles and associated muscles of the lower urinary tract, which may lead to UI or dysuria.

Sarcopenia is associated with impaired detrusor muscle contraction function (94). Furthermore, a study has pointed out that sarcopenia may be related to autonomic dysfunction (95). And abnormal autonomic function controlling the bladder may affect detrusor muscle contraction (96). Detrusor muscle contraction disorders may lead to secondary symptoms such as difficulty urinating, urinary retention, and overflow incontinence (1). Furthermore, abnormal detrusor muscle function, whether overactivity or underactivity, is an important mechanism in the development of OAB and may contribute to its occurrence (96). However, there is still a relative lack of research that directly proves sarcopenia affects the detrusor muscle of the bladder by influencing the autonomic nervous system. Further research in this area is needed.

### 3.3 Metabolic and endocrine disorders

Current research has revealed a significant link between sarcopenia and metabolic and endocrine disorders. Androgens are significantly associated with the occurrence of sarcopenia. Studies have shown that in male patients undergoing hemodialysis, serum free testosterone levels are significantly associated with sarcopenia, and low free testosterone levels are also associated with a decrease in muscle mass (97). Patients undergoing ADT experience a significant decrease in muscle mass after treatment, suggesting that a decrease in androgen levels may lead to the development of sarcopenia (59, 66, 98). Furthermore, ADT-associated loss of lean mass may be selective, with the levator ani muscle potentially experiencing more significant loss. This suggests that androgen deficiency may be involved in the association between sarcopenia and LUTDs by weakening the function of pelvic floor muscles (such as the levator ani muscle) (99).

Furthermore, sarcopenia may be related to metabolic disorders. Sarcopenia, as a manifestation of cancer cachexia, reflects more severe metabolic disorders and inflammatory states in patients. Patients with sarcopenia have an increased risk of complications related to malignant tumor treatment and a poorer prognosis (100). Research using multi-omics mediational analysis has shown that 17 metabolites and proteins may play a potential mediating role between sarcopenia and BPH and acute tubulointerstitial nephritis. These substances reflect a metabolic interdependence between sarcopenia and urological diseases, but no significant immune mediators were identified (72).

### 3.4 Physical activity restriction and genetics

Physical activity restriction caused by sarcopenia may be a cause of LUTS. Sarcopenia can slow down walking speed. Studies show that walking speed is the only independent risk factor for UI (HR = 0.05, 95% CI 0.003–0.987, *p* = 0.049), meaning that the slower the walking speed, the higher the risk of UI (101). The reason for this might be that slower walking speed makes it harder to get to the bathroom. In addition, sarcopenia is often accompanied by other symptoms of physical weakness. An increase in individual weakness scores is associated with a non-linear increase in the severity of LUTS (102).

In addition to acquired factors, genetic factors may also play a role in the association of sarcopenia with LUTDs. Yang et al. identified a genetic comorbidity between sarcopenia and BPH through multiple analytical methods. In a specific region of chromosome 3 (3:139954597–141,339,097), the two conditions exhibit local genetic association (*p* = 2.16 × 10^−5^). In cell culture fibroblast tissues, the heritability of sarcopenia and BPH was enriched. The study also identified 75 shared risk genes for sarcopenia and BPH, which were enriched in functional pathways such as cellular component biogenesis, RNA binding, metabolic pathways, fatty acid metabolism, and biotin metabolism (72). These multi-omic correlations motivate causal inference and tissue-specific functional validation.

### 3.5 Gut microbiome

Gut microbiome also plays an important role in sarcopenia and LUTDs. In sarcopenia, animal models and human studies have consistently shown a significant increase in the abundance of Bacteroides, Clostridia, and a decrease in the abundance of Short-Chain Fatty Acids (SCFA)-producing beneficial bacteria (e.g., *Lactobacillus*, *Prevotella*) in sarcopenic patients (103–108). A review study concluded that the gut microbiome affects muscle health in two main ways: 1. dysbiosis leads to disruption of the gut barrier and chronic inflammation throughout the body, which leads to a decrease in muscle mass and strength; 2. dysbiosis leads to the production of deleterious metabolites, which leads to muscle atrophy and dysfunction; and a decrease in beneficial metabolites leads to further deterioration of muscle function (109).

Similarly, the gut microbiome is closely related to LUTDs. It has been noted that the composition of the intestinal flora of patients with OAB and daily urinary urgency is significantly different from that of the healthy population (110); even more, studies have shown that the characterization of baseline gut flora can predict the progression of future OAB symptoms (111). Dysbiosis in the gut microbiome also contributes to the metabolic activation of precarcinogens such as nitrosamines. These substances are excreted in urine and, through prolonged contact with bladder epithelium, increase the risk of carcinogenesis. It also drives a state of inflammation that collectively promotes the development of bladder cancer (112, 113). And several studies have used genetic methods to confirm that there is a causal relationship, not just a correlation, between specific gut flora and prostate cancer risk (114).

All of the above findings suggest that the gut microbiome is associated with both sarcopenia and LUTDs, and that improving dysregulation of the gut microbiome could be a therapeutic target for improving both sarcopenia and LUTDs.

In summary, the association between sarcopenia and LUTDs is not the result of a single mechanism, but is mediated through a multidimensional pathway of pelvic floor muscle and urethral sphincter weakening, neuromuscular dysfunction, metabolic disorders, limited physical activity, genetic factors, or the gut microbiome, ranging from insufficient structural muscle support, to impaired neural signaling, to indirect effects of intestinal bacteria through inflammatory and metabolic pathways. These mechanisms not only reveal the complexity of the interaction between the two, but also identify the core targets for clinical intervention - whether it is repairing muscle function, regulating metabolic status, or improving intestinal microecology, all of them should be centered on the key links in the mechanisms. Based on the scientific support of the above mechanisms, intervention for LUTDs has become an important breakthrough to improve the symptoms and optimize the prognosis of LUTDs, and a number of intervention strategies have already demonstrated their potentials in clinical research, covering the fields of exercise training, nutritional regulation, intestinal bacterial targeting intervention, local muscle rehabilitation, and pharmacological treatment, etc. In the following section, we will systematically elaborate the specific implementation plan, effect and clinical application value of these strategies. In the following, we will systematically describe the implementation, effect and clinical value of these strategies.

## 4 Clinical implications of sarcopenia-targeted therapies in the management of lower urinary tract diseases

**Figure 2:**
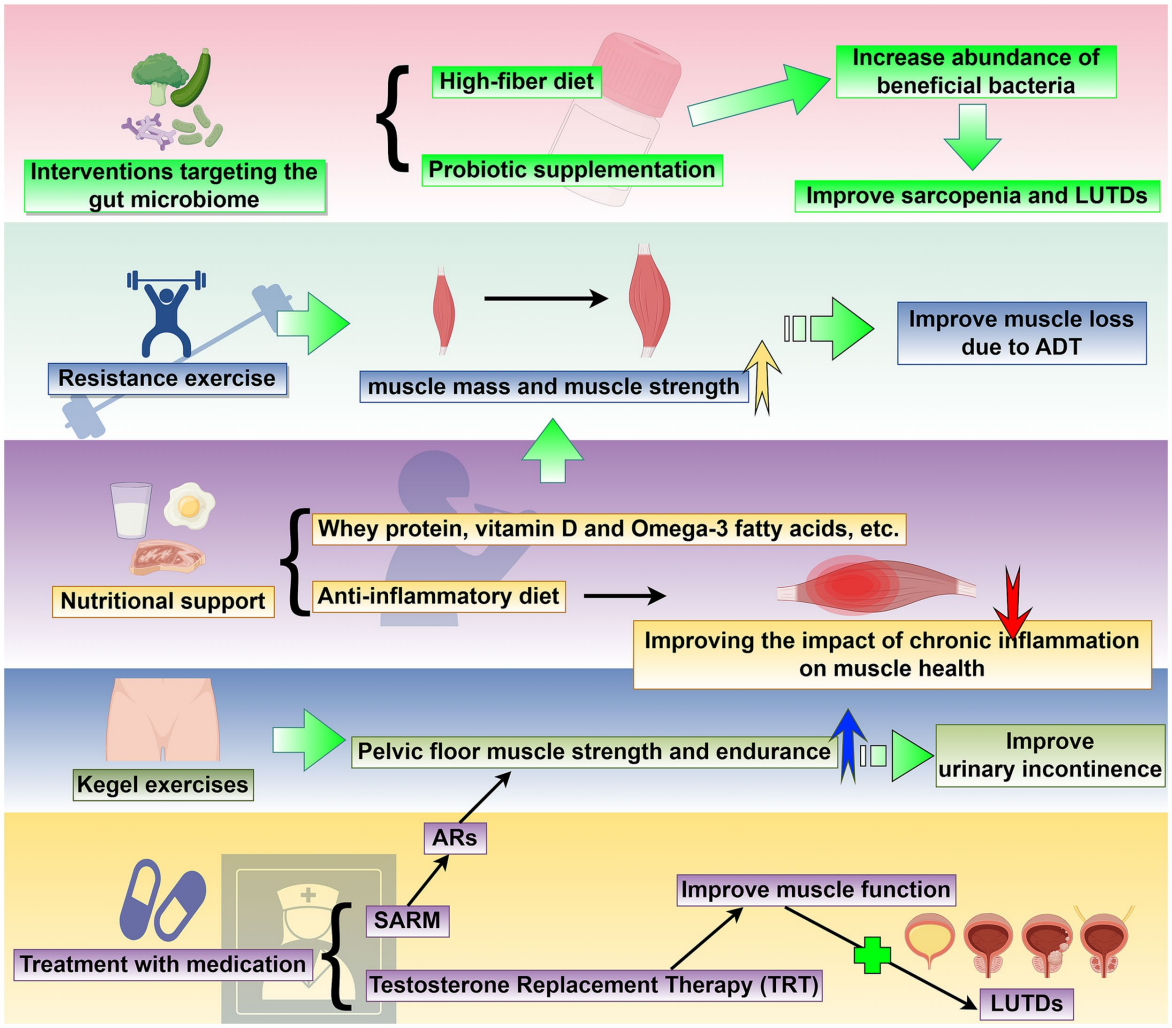
Targeted sarcopenia therapy for potential treatment methods of LUTDs: This schematic outlines a comprehensive set of strategies to address sarcopenia and its associated LUTDs. The interventions include: (1) resistance exercise: Progressive resistance training enhances muscle mass and strength, effectively reversing muscle loss associated with androgen deprivation therapy (ADT) and serving as a non-pharmacological approach to mitigate LUTD-related dysfunction. (2) Nutritional support: Supplementation with whey protein, vitamin D, omega-3 fatty acids, and adherence to an anti-inflammatory diet improve muscle health by countering chronic inflammation and promoting muscle synthesis, with implications for LUTD management. (3) Interventions targeting the gut microbiome: High-fiber diets and probiotic supplementation increase the abundance of beneficial bacteria, modulating systemic inflammation and metabolic pathways to concurrently improve sarcopenia and LUTDs. (4) Kegel exercises: Pelvic floor muscle training strengthens pelvic floor muscle strength and endurance, directly improving urinary incontinence and other LUTD symptoms. (5) Treatment with medication: Pharmacological strategies, including selective androgen receptor modulators (SARMs), androgen receptor (AR)-targeted agents, and testosterone replacement therapy (TRT), enhance muscle function by addressing androgen-related sarcopenia, subsequently alleviating LUTDs. Collectively, these interventions target sarcopenia through multiple pathways, with the potential to concurrently improve LUTD outcomes and enhance patient quality of life. This figure was drawn by Figdraw.com.

A multifaceted approach targeting muscle preservation, functional improvement, and symptom alleviation is required to manage sarcopenia-related LUTDs. There is emerging evidence to support the efficacy of resistance exercise, nutritional interventions, Interventions targeting the gut microbiome, pelvic floor training and pharmacological therapies in addressing the underlying muscle loss and its associated urinary complications (Figure 2). This section summarizes current therapeutic strategies, emphasizing their clinical applications and potential to improve the quality of life of affected patients (Table 2). Future research should focus on personalized regimens to optimize outcomes.

**Table 2:** Intervention strategies and clinical implications.

Resistance exercise	Nutritional support	Interventions targeting the gut microbiome	Kegel exercises	Treatment with medication
(1) Effectively increasing muscle mass and strength; (2) Improving lean body mass in PCa patients undergoing ADT.	(1) Effectively increasing muscle mass and strength; (2) Improving patients’ clinical outcomes, especially those with cancer.	(1) Effectively increasing muscle mass and strength; (2) Reducing the recurrence rate of malignant tumors.	Strengthening pelvic floor muscle strength and significantly improving UI.	Improving UI and LUTS.

PCa, prostate cancer; ADT, androgen deprivation therapy; UI, urinary incontinence; LUTS, lower urinary tract symptoms.

### 4.1 Resistance exercise

Recent studies have shown that resistance exercise can significantly improve sarcopenia and increase muscle strength. Multiple randomized controlled trials have confirmed that regular resistance exercise can effectively increase muscle mass and strength (115). Intensive Lifestyle Intervention (Whey Protein Supplementation + Resistance Training) Leads to Significant Improvements in Muscle Mass in Patients with sarcopenia (116). Especially for prostate cancer (PCa) patients undergoing androgen deprivation therapy (ADT), resistance exercise for more than 12 weeks can significantly reverse ADT-associated loss of lean mass (117, 118). The mechanism of action may be that resistance exercise significantly increases MuRF-1 mRNA expression, and changes in MuRF-1 mRNA expression are associated with improvements in muscle strength and function (118). In terms of exercise programs, the study recommends progressive load training 2–3 times a week, combined with protein supplementation to enhance muscle synthesis (115, 119–121). It is worth noting that research by Galvão et al. shows that a combination of resistance exercise and aerobic exercise also has excellent therapeutic effects (121). However, neither resistance training nor aerobic training should be too intense, as studies have shown that high-intensity exercise (more than 8 h per week) can lead to an increased incidence of urinary incontinence (UI) (122). Therefore, maintaining the appropriate intensity is equally important. Based on the above evidence, intensity-appropriate resistance exercise for sarcopenia is a safe, cost-effective, and effective intervention for the treatment of lower urinary tract dysfunction associated with sarcopenia as a nonpharmacologic treatment option. However, current literature remains markedly deficient in direct empirical research demonstrating whether resistance exercise can simultaneously improve sarcopenia and LUTDs. Further investigation into the specific role of resistance exercise in managing LUTDs holds significant translational value for advancing clinical practice. Such research may fill critical evidence gaps, clarifying whether resistance exercise—as a targeted intervention for sarcopenia—can serve as a viable non-pharmacological strategy for alleviating LUTDs.

### 4.2 Nutritional support

Nutritional support targeting sarcopenia is of great significance in the management of LUTDs. Research indicates that sarcopenia is a key biomarker for bladder cancer (BC) prognosis (123). A decrease in psoas muscle mass and nutritional index after surgery significantly affects patient clinical outcomes (124). Inadequate protein intake may be associated with sarcopenia in older adults (125). A healthy diet can slow the decline in physical performance (126). For elderly patients, a combination of nutritional supplements containing whey protein, vitamin D and omega-3 fatty acids and resistance training can effectively improve muscle quality and strength (115). Results from a randomized clinical trial showed that 12 consecutive weeks of daily supplementation with a high-protein oral supplement containing 3 grams of HMB (Ensure® Plus Advance) significantly increased muscle mass (thigh thigh cross-sectional area), body weight, and body mass index in frail older adults (127). However, the components of an anti-inflammatory diet may help to alleviate the impact of chronic low-grade inflammation on muscle health (128). In addition, fruit and vegetable intake may also reduce the risk of sarcopenia (129). In perioperative management, preoperative optimization for patients with genitourinary tumors should include nutritional assessment and support (130). Furthermore, the cachexia state of late-stage cancer patients is closely related to their quality of life and requires comprehensive nutritional intervention (131, 132). In clinical practice, it is recommended that patients with LUTDs, especially the elderly and cancer patients, undergo routine screening for sarcopenia and nutritional assessment to develop personalized nutritional support programs (123, 133, 134). However, similar to resistance training, the majority of research evidence concerning nutritional support primarily supports its role in improving sarcopenia. Direct evidence regarding nutritional support’s direct improvement of LUTDs remains lacking. Therefore, further investigation into the specific role of nutritional support in managing LUTDs holds significant value and provides practical guidance for formulating patient treatment strategies.

### 4.3 Interventions targeting the gut microbiome

Improving the gut microbiome through dietary modifications, nutritional interventions, and probiotic supplementation has great potential in the treatment of sarcopenia and LUTDs. Dietary protective effects on skeletal muscle need to be mediated through the gut microbiota; however, gut dysbiosis is common in the elderly population, and therefore, the design of nutritional strategies for sarcopenia needs to focus on the role of gut flora (135). And to improve the ratio of the gut microbiome, a high-fiber diet is especially important. One study suggests that the Mediterranean diet may maintain or even enhance flora diversity (136). Dietary fiber increases the relative abundance of *bifidobacteria* (137). An animal study finds that a high-fiber diet upregulates *Bifidobacterium pseudolongum* and *Lactobacillus johnsonii* abundance (138). This evidence suggests that diets high in dietary fiber may increase the proportion of beneficial intestinal bacteria and therefore also ameliorate sarcopenia and LUTDs, but gaps in relevant experimental evidence remain.

In addition to a high-fiber diet, direct probiotic supplementation improves the gut microbiota and can increase muscle mass and overall muscle strength (139). Supplementation with *Lactobacillus casei Shirota* reduced age-related declines in muscle mass, strength and mitochondrial function (140). And there are also studies that suggest that supplementation with *Lactobacillus casei* probiotics can reduce non-muscle invasive BC (NMIBC) recurrence (141). Therefore, targeting the gut microbiome to ameliorate sarcopenia and delayed-onset muscular dystrophy has great potential.

### 4.4 Kegel exercises

Kegel exercises (Pelvic Floor Muscle Training, PFMT) involve strengthening the pelvic floor muscles, which can significantly improve symptoms of UI. This has been supported by multiple clinical studies (142, 143). Studies have shown that Kegel exercises increase the strength and endurance of the pubococcygeus muscle, which improves the ability of the urethral sphincter to close, which could prove that Kegel exercises reduce the frequency of stress urinary incontinence (SUI) and mixed urinary incontinence (MUI) (142–145). For example, a randomized controlled trial found that home Kegel exercises significantly improved the quality of life of women with SUI and reduced the frequency of incontinence episodes (143). In addition, combining Kegel exercises with biofeedback can further optimize the exercise effect, and real-time monitoring of muscle contraction helps patients master the correct technique (146–148). Long-term follow-up studies show that patients who persist with Kegel exercises experience sustained relief from UI symptoms (149). Therefore, Kegel exercises, as a non-invasive, low-cost intervention, can specifically strengthen the pelvic floor muscles and significantly improve UI symptoms.

### 4.5 Treatment with medication

Selective androgen receptor modulators (SARMs) are a class of small-molecule drugs that act as agonists or antagonists of the androgen receptor (AR), mimicking the anabolic effects of testosterone. They selectively target specific tissues, including muscle, due to differences in AR regulatory proteins in these tissues (150). After entering the cytoplasm through the cell membrane, SARMs bind to (androgen receptors) ARs. The resulting SARM-AR complex then translocates to the cell nucleus. Within the nucleus, this complex exerts transcriptional regulatory effects by selectively recruiting different co-regulatory proteins and transcription factors to regulate the expression of downstream target genes (151). Research evidence suggests that SARMs, such as GTx-024/enobosarm and its structural analog GTx-027, demonstrate significant therapeutic potential in conditions such as sarcopenia and SUI (152). Their mechanism of action may involve anabolic regulation of the pelvic floor muscle group (particularly the levator ani muscle): animal studies have shown that SARMs can selectively activate ARs, thereby promoting an increase in levator ani muscle mass and contractile function, and improving the supportive function of the urethral sphincter system (152, 153). In summary, SARMs represent a promising therapeutic approach for sarcopenia and SUI, as they selectively enhance pelvic muscle function through targeted AR activation.

Testosterone replacement therapy (TRT) is a medical treatment for hypogonadism in men. It involves supplementing exogenous testosterone to restore normal serum testosterone levels and improve symptoms caused by testosterone deficiency. In recent years, the application of TRT has expanded, but it has also been accompanied by controversy, especially in the fields of anti-aging and performance enhancement. Research has shown that testosterone deficiency is associated with sarcopenia (154). A study of elderly men showed that TRT can improve muscle function (155). Although there is no research evidence to support this, we can speculate that improving muscle condition through TRT could alleviate lower urinary tract symptoms (LUTS) caused by sarcopenia and improve quality of life. Future research should focus on exploring safer administration methods and precise, individualized treatment.

Although current medications show promise in treating sarcopenia-related LUTS, more research is needed to improve their target specificity and safety profiles. Future studies should explore additional drug candidates and develop personalized treatment strategies using precision medicine approaches.

## 5 Conclusion and perspectives for future research

A growing body of evidence indicates that sarcopenia is both a consequence and a driver of lower urinary tract symptoms (LUTDs). Our analysis confirms that sarcopenia impacts the severity, treatment response, and clinical outcomes of multiple key urological conditions, including lower urinary tract symptoms (LUTS), overactive bladder (OAB), urinary incontinence (UI), bladder cancer (BC), prostate cancer (PCa), and benign prostatic hyperplasia (BPH).

Emerging interventions for sarcopenia—such as resistance training, nutritional support, gut microbiota modulation, and pharmacotherapy—hold promise for breaking the vicious cycle between muscle loss and LUTD progression. However, the existing evidence base has critical limitations: some studies are too small, resulting in weak evidence strength. Moreover, most studies employ cross-sectional or retrospective designs, with widespread methodological heterogeneity. For instance, diagnostic criteria for sarcopenia vary across studies—despite the 2019 EWGSOP2 consensus requiring comprehensive assessment of muscle mass, strength, and function, many studies rely on single indicators (e.g., iliopsoas index) or unvalidated measurement methods. This creates three major issues: inconsistent standards impede validation of mechanistic hypotheses; and imprecise patient enrollment confounds interpretation of intervention effects, ultimately undermining treatment credibility.

We strongly recommend integrating sarcopenia screening into routine urological care—early detection and intervention can significantly improve patient outcomes due to sarcopenia’s reversibility. Multidisciplinary collaboration is essential, requiring the combined expertise of urology, geriatrics, nutrition, and rehabilitation teams to address sarcopenia’s systemic impact and optimize treatment strategies for lower urinary tract dysfunction (LUTDs). Future research should focus on three key directions: Conducting large-scale, standardized prospective trials using unified sarcopenia diagnostic criteria (following the EWGSOP2 guidelines) to validate the causal relationship between sarcopenia and LUTDs and establish evidence-based management protocols; Mechanism Elucidation: Focusing on subtype-specific pathways to identify novel therapeutic targets; Developing personalized interventions: Tailoring precise intervention strategies based on LUTD subtypes and patient characteristics (e.g., age, comorbidities, baseline muscle status) to optimize treatment outcomes. By addressing these research gaps, we will advance care models centered on sarcopenia within LUTD management, ultimately enhancing quality of life for an increasingly large patient population.
